# Three-Day Continuous Oxytocin Infusion Attenuates Thermal and Mechanical Nociception by Rescuing Neuronal Chloride Homeostasis *via* Upregulation KCC2 Expression and Function

**DOI:** 10.3389/fphar.2022.845018

**Published:** 2022-03-24

**Authors:** Xiyuan Ba, Chenqiu Ran, Wenjun Guo, Jing Guo, Qian Zeng, Tao Liu, Wuping Sun, Lizu Xiao, Donglin Xiong, Yelan Huang, Changyu Jiang, Yue Hao

**Affiliations:** ^1^ Department of Pain Medicine and Shenzhen Municipal Key Laboratory for Pain Medicine, Shenzhen Nanshan People’s Hospital, Shenzhen, China; ^2^ School of Pharmaceutical Sciences, Health Science Center, Shenzhen University, Shenzhen, China; ^3^ Department of Pain Medicine, Shenzhen, China; ^4^ Department of Endocrinology and Metabolism, Shenzhen University General Hospital and Shenzhen University Academy of Clinical Medical Sciences, Shenzhen University, Shenzhen, China; ^5^ Department of Pediatrics, The First Affiliated Hospital of Nanchang University, Nanchang, China

**Keywords:** neuropathic pain, oxytocin, chloride homeostasis, K+-Cl-cotransporter 2, continuous intrathecal drug delivery

## Abstract

Oxytocin (OT) and its receptor are promising targets for the treatment and prevention of the neuropathic pain. In the present study, we compared the effects of a single and continuous intrathecal infusion of OT on nerve injury-induced neuropathic pain behaviours in mice and further explore the mechanisms underlying their analgesic properties. We found that three days of continuous intrathecal OT infusion alleviated subsequent pain behaviours for 14 days, whereas a single OT injection induced a transient analgesia for 30 min, suggesting that only continuous intrathecal OT attenuated the establishment and development of neuropathic pain behaviours. Supporting this behavioural finding, continuous intrathecal infusion, but not short-term incubation of OT, reversed the nerve injury-induced depolarizing shift in Cl^−^ reversal potential *via* restoring the function and expression of spinal K^+^-Cl^-^ cotransporter 2 (KCC2), which may be caused by OT-induced enhancement of GABA inhibitory transmission. This result suggests that only continuous use of OT may reverse the pathological changes caused by nerve injury, thereby mechanistically blocking the establishment and development of pain. These findings provide novel evidence relevant for advancing understanding of the effects of continuous OT administration on the pathophysiology of pain.

## Introduction

Neuropathic pain is a debilitating condition that affects 7–10% of the general population (Colloca et al., 2017). Unlike opioids and non-steroidal anti-inflammatory drugs for nociceptive pain, the medications used to treat neuropathic pain tend to only be modestly effective and can potentially cause multiple adverse reactions (Baron et al., 2010). Developing mechanism-based therapies for neuropathic pain remains a major challenge. A growing body of literature has demonstrated the analgesic effects of the neuropeptide oxytocin (OT) in both humans and rodents (see reviews by Oxytocin and pain perception: from animal models to human research) (Gimpl and Fahrenholz, 2001; Honda and Takano, 2009; Koshimizu and Tsujimoto, 2009; Stoop, 2014; Boll et al., 2018; Herpertz et al., 2019). Electrical stimulation of the anterior part of the hypothalamic paraventricular nucleus increased OT concentration in the cerebrospinal fluid (CSF) and produced antinociception in rats (Martinez-Lorenzana et al., 2008), and intraperitoneal or intrathecal (i.t.) injection of OT was shown to block neuropathic pain in rats (Yang et al., 2007). Clinical data suggested that administration of OT in the cerebrospinal fluid (CSF) reduces surgical recovery time while decreasing pain and hypersensitivity in patients after injury (Wang et al., 2013). Considering it also plays a key modulatory role in emotions, stress and anxiety, which are well known to substantially influence pain perception (Apkarian et al., 2005; Apkarian, 2008; Baron et al., 2010; Peters, 2015; Tracy et al., 2015), OT has become a promising target for therapeutic interventions for pain.

Excitation/inhibition imbalance along the entire nociceptive pathway is considered a main driver in the development of neuropathic pain (Kahle et al., 2014). One of the mechanisms proposed for this imbalance involves compromised inhibition in the superficial dorsal horn of the spinal cord, leading to hyperactivity of spinal dorsal horn circuit, which is the main target for primary nociceptive afferents (Prescott, 2015). γ-aminobutyric acid (GABA) is the most critical inhibitory neurotransmitter in the central nervous system. The inhibitory efficiency of GABAergic transmission is determined primarily by the electrochemical gradient for Cl^−^, which is depended by the intra and extracellular concentration of Cl^−^ (Ganguly et al., 2001). It has been demonstrated that Cl^−^ homeostasis is collapsed and Cl^−^ levels are elevated in spinal cord neurons under the pathophysiology of pain disorders (Coull et al., 2003). Recently, a body of evidence showed that compromised spinal inhibition resulted from downregulation of K^+^-Cl^-^ cotransporter 2 (KCC2) and the subsequent disruption of intracellular chloride homeostasis (Coull et al., 2003; Price et al., 2009; Li et al., 2016; Mapplebeck et al., 2019). In mature central neurons, KCC2 is responsible for the low intracellular Cl^−^ concentration ([Cl^−^]_i_) that forms the basis for hyperpolarizing GABA_A_ receptor-mediated responses. It regulates the formation (Li et al., 2007), functional maintenance and plasticity of glutamatergic synapses (Fiumelli et al., 2005; Gauvain et al., 2011; Chevy et al., 2015; Llano et al., 2015). Indeed, Modol’s results indicate that nerve injury results in a reduction in the expression of KCC2 in the spinal dorsal horn that accompanies chronic pain, but prevention of the downregulation of KCC2 along the central sensory pathways relieves neuropathic pain after peripheral nerve injury (Modol et al., 2014). Loss of activity of this transporter is a key mechanism for chronic pain, and different groups demonstrated that renormalization of impaired KCC2 alleviated nerve injury-induced neuropathic pain (Gagnon et al., 2013; Kitayama, 2017). Leonzino et al. found that OT directly modulates the functional activity of KCC2 by promoting its phosphorylation and insertion/stabilization at the neuronal surface in an early developmental time window (Leonzino et al., 2016). However, little is known on how OT affects chloride homeostasis and the function of KCC2 in neuropathic pain.

In addition, the current understanding of mechanisms underlying OT analgesia is mainly based on studies using single or multiple injections of OT in animals. Little is known about the effects of continuous OT administration on pain processing. In this study, we adopted intrathecal drug delivery technique to administer OT centrally in nerve injured mice. Chronic intrathecal drug infusion through an implantable pump is a clinically available strategy to treat a number of neurological diseases (Ganguly et al., 2001; Kästner, 2010). Findings based on continuous intrathecal OT delivery in mice may provide more information on how OT targets the pathophysiology of pain and better implications for human therapy.

Thus, in the present study we adopted intrathecal drug delivery technique to compare the effects of a single or continuous intrathecal infusion of OT on pain behaviours in mice; we determined whether they block neuropathic pain by preventing the disruption of the intracellular Cl^−^ homeostasis in the spinal superficial dorsal horn, and whether it is mediated by restoring the KCC2 expression and function.

## Materials and Methods

### Animals

All animal procedures were conducted in strict adherence to the guidelines of the International Association for the Study of Pain and were approved by the Animal Care and Use Committee of Health Science Center at Shenzhen University. 80 male C57BL/6 mice (5–8 weeks of age) were purchased from Guangdong Province Laboratory Animal Center (Guangzhou, China). 20 vGAT-ires-cre mice and 20 td-Tomato (Ai9) mice were purchased from Jackson Laboratory. The animals were housed in plastic cages (5 per cage) in a temperature-controlled environment on a 12 h/12 h light/dark cycle. Food and water were available *ad libitum*.

### Reagents

Oxytocin (catalogue: H-2510) and [d(CH2)51,Tyr(Me)2, Thr4,Orn8,des–Gly–NH29]–vasotocin (dVOT, catalogue: H-2510) were purchased from Bachem AG (Bubendorf, Switzerland). TC OT39 (catalogue: 1078) was obtained from Tocris (Minnesota, United States).

### Neuropathic Pain Model

The partial sciatic nerve ligation (pSNL) pain model was established according to previously described procedures (Seltzer et al., 1990). Briefly, the animals were anaesthetized with sodium pentobarbital (50 mg/kg, i.p.) and a tight ligation of approximately one-third to one-half the diameter of the right sciatic nerve (ipsilateral) was performed with 6–0 silk suture. In sham-operated mice, the nerve was exposed without ligation.

### Behavioural Testing

Von Frey testing was performed to assess mechanical allodynia. The mice were habituated to the environment for 2 days before the testing began. All the behaviours were tested blindly. For testing mechanical allodynia, the mice were confined separately in boxes (14 × 18 × 12 cm) placed on an elevated metal mesh floor, and their hind paws were stimulated with a series of von Frey hairs with logarithmically increasing stiffness (0.16–2.00 g, Stoelting) situated perpendicularly to the central plantar surface. The 50% paw withdrawal threshold was determined by Dixon’s up-down method. The hot plate test (Hot/Cold Plate, Cat. 35150, Ugo Basile, Italy) was used to examine thermal hyperalgesia. Each mouse was placed on the hot plate, and the latency of paw withdrawal from the heat stimulus was measured twice separated by a 5-min interval. The average value was used as the latency of response. All behavioural testing was done with the experimenters blinded to the treatment conditions.

### Intrathecal Injection and Continuous Intrathecal Infusion of Drugs

OT (0.1 μg in 10 μL) or dVOT (0.1 μg/10 μL) was injected into the subarachnoid space through the intervertebral foramen between L4 and L6 (Hylden and Wilcox, 1980). For the intrathecal infusion of drugs, an osmotic minipump (model 1003D, ALZET, Cupertino, CA, United States) connected with a polyethylene catheter was deposited in a subcutaneous pocket following partial sciatic nerve ligation. The other end of the catheter was inserted from the atlanto-occipital membrane into the subarachnoid space until the tip of the catheter reached the lumbar spinal enlargement. OT and other reagents were then delivered continuously with a flow rate of 1 μL/h for 3 days from days 0 to 2 after pSNL surgery. The final dose of OT intrathecal infusion is 0.3 μg in 100 μL. (The volume delivery rate and the delivery duration of ALZET pumps are fixed at manufacture).

### Quantitative RT-PCR

The animals were sacrificed and L4–6 spinal cord segments were collected in tubes with RNAlater (Qiagen Inc., Valencia, CA, United States) and stored at −80°C until RNA isolation. Total RNA was isolated from these tissues according to Chomczynski’s method (Chomczynski and Sacchi, 1987) and reverse transcribed using Omniscript reverse transcriptase (Qiagen Inc., Valencia, CA, United States) at 37°C for 60 min. The reaction was performed in the presence of the RNase inhibitor rRNAsin (Promega, Madison, WI, United States) and an oligo (dT16) primer (Qiagen) to selectively amplify the mRNA. For quantitative PCR, 45 ng of cDNA was used as a template. Reactions were performed using Assay-On-Demand TaqMan probes and TaqMan Universal PCR Master Mix (Applied Biosystems, Foster, CA, United States) according to the manufacturer’s protocol. Reactions were run on a Real-Time PCR iCycler IQ (Bio-Rad, Hercules, CA, United States) with software version 3.0. The expression levels of *Kcc2* were normalized to *ß*-actin.

### Western Blotting

The animals were sacrificed, and the L4-6 spinal cord segments were removed and stored at −80°C until assayed. The samples were homogenized and centrifuged to extract the protein, and the resulting preparations were saved. Equal amounts of protein were separated by 10% Tris-Tricine SDS-PAGE and transferred onto polyvinylidene difluoride membranes. The membranes were then blocked in 5% non-fat milk for 1 h at room temperature, followed by overnight incubation with rabbit anti-KCC2 antibody (1:1000; ab49917, Abcam, United States) and *ß*-actin (1:2000; Sigma, United States) primary antibody. Immunoblots were then incubated for 1 h at room temperature with goat anti-rabbit polyclonal IgG (1:3000, ab205718, Abcam, MA, United States). Immunoblots were developed by chemiluminescent substrate and quantified using ImageJ software.

### Immunohistochemistry

The mice were deeply anesthetized with isoflurane and transcardially perfused with PBS followed by 4% PFA. Lumbar L4-6 spinal cord segments sections were blocked and then incubated overnight at 4°C with rabbit antibodies against KCC2 (Abcam, ab49917, United States). The sections were then incubated for 30 min at 37°C with AF488-conjugated secondary antibodies (donkey, 1:500, Jackson Immuno-Research, West Grove, PA, United States), and the nuclei were stained with DAPI. The sections were viewed under Zeiss 880 inverted confocal microscopy, and images were collected using identical acquisition parameters and quantified using Image-Pro Plus 6.0 software (Media Cybernetics, Silver spring, MD, United States) by experimenters blinded to treatment groups.

### *In Situ* Hybridization

*In situ* hybridization was performed using the RNAscope system (Advanced Cell Diagnostics) following the manufacturer’s protocol. Pre-treatment consisted of dehydration, followed by incubation with hydrogen peroxide and protease IV at room temperature. The Multiplex Fluorescent Kit v2 protocol was followed using commercial probes for the OT receptor (Oxtr, NM_001081147.1, #402658-C3). Images were captured by Zeiss 880 inverted confocal microscopy. Visualized cells with more than 5 puncta per cell were classified as positive neurons.

### Electrophysiological Recordings

Adult (5–7 weeks) male mice were anaesthetized with urethane (1.5–2.0 g/kg, i.p.). The lumbosacral spinal cord was removed and submerged into ice-cold dissection solution saturated with 95% O_2_ and 5% CO_2_ at room temperature. Transverse slices (300–400 μm) were cut in a vibrating microslicer (VT1200s Leica). The slices were incubated at 32°C for at least 30 min in regular artificial cerebrospinal fluid (aCSF) equilibrated with 95% O_2_ and 5% CO_2_.

The following solutions were used: dissection solution containing (in mM) 240 sucrose, 25 NaHCO_3_, 2.5 KCl, 1.25 NaH_2_PO_4_, 0.5 CaCl_2_, and 3.5 MgCl_2_ at pH 7.4; regular artificial CSF containing 135 NaCl, 2.5 KCl, 3 MgCl_2_, 1 CaCl_2_, 10 HEPES, 1 NaH_2_PO_4_, and 10 glucose at pH 7.4; and normal intrapipette solution for perforated recording containing 115 K-methylsulfate, 25 KCl, 2 MgCl_2_, 10 HEPES, 0.4 GTP-Na and 5 Mg-ATP at pH 7.2 and 310 mOsm.

To measure the reversal potential of GABA-evoked currents, a slice was placed in the recording chamber and completely submerged and superfused at a rate of 2–4 ml/min with aCSF. A perforated patch-clamp was applied to avoid changes in the [Cl^−^]_i_. To measure the chloride equilibrium potential (E_Cl_), gramicidin D (80 μg/ml with an 0.8% DMSO final concentration from an 8 mg/ml stock in DMSO) was added to the intrapipette solution, and 6-cyano-7- nitroquinoxaline-2,3-dione (CNQX, 10 μM), DL-2-amino-5-phosphonovaleric acid (APV, 50 μM) and tetrodotoxin (TTX, 0.5 μM) were added to the aCSF solution. The tip of the patch pipette was filled with the normal intrapipette solution, while the rest of the pipette contained the gramicidin-containing solution. After forming a seal on the membrane, we waited 30 min for the gramicidin to effectively reduce the series resistance to below 100 MΩ. Membrane potential measurements were corrected for liquid junction potential, which was measured as in(Guo et al., 2014). GABA (1 mM) was puffed locally and instantaneously, and the puff pipette was aimed toward the recording pipette. Voltage ramps were applied from +8 to −92 mV over 200 ms at a holding potential of −42 mV. Since the voltage ramp might evoke a basal current, a control voltage ramp was first applied to record the basal current; 1 min later, GABA was puffed, followed by another voltage ramp, and then the GABA-evoked currents were recorded (Billups and Attwell, 2002). The reversal potential was analysed as in (Billups and Attwell, 2002).

Excitatory and inhibitory post-synaptic currents (EPSCs and IPSCs) recordings were made from lamina II inhibitory neurons. The patch-pipette solution contained (in mM) K-gluconate 135, KCl 5, CaCl_2_ 0.5, MgCl_2_ 2, EGTA 5, HEPES 5, an Mg-ATP 5; or Cs_2_SO_4_ 110, CaCl_2_ 0.5, MgCl_2_ 2, EGTA 5, HEPES 5, Mg-ATP5, tetraethylammonium (TEA)-Cl 5 (pH = 7.2) (Jiang et al., 2014). The former and latter solutions were used to record EPSCs and IPSCs, respectively. EPSC recordings were made at a holding potential (V_H_) of −70 mV, where no IPSCs were observed, since the reversal potential for IPSCs was near −70 mV. IPSCs were recorded at a V_H_ of 0 mV, where EPSCs were invisible as reversal potential for EPSCs was close to 0 mV. Cs^+^ and TEA were used to block K^+^ channels expressed in the recorded neurons, and thus to easily shift V_H_ from −70 to 0 mV. GABAergic IPSCs were obtained in the presence of the glycine-receptor antagonist strychnine (1 mM). EPSC and IPSC events were detected and analysed using Mini Analysis Program 6.0. Signals were acquired using an Axopatch 700B amplifier and analysed with pCLAMP 10.3 software. Only neurons with resting membrane potential < −50 mV and stable access resistance were included.

### Statistical Analysis

The data are expressed as means ± SEM and analysed with a *t*-test or variance (ANOVA) using one-way or mixed factorial designs as appropriate, followed by Bonferroni’s *post hoc* test or simple-effects ANOVA. All statistical analyses were performed using GraphPad Prism 8.0. (GraphPad Inc., La Jolla, CA, United States). Significance was defined as *p* < 0.05.

## Results

### Three-Day Continuous Intrathecal Infusion, but Not Short-Term Application of OT, Attenuated the Establishment and Development of Nerve Injury-Induced Nociceptive Behaviours in pSNL Mice

pSNL-induced nerve injury produced mechanical allodynia and thermal hyperalgesia in mice. This mechanical and thermal hypersensitivity started on day 1 and remained relatively stable from days 3 to 14 after nerve ligation (Supplementary Figures S1A,B).

**FIGURE 1:**
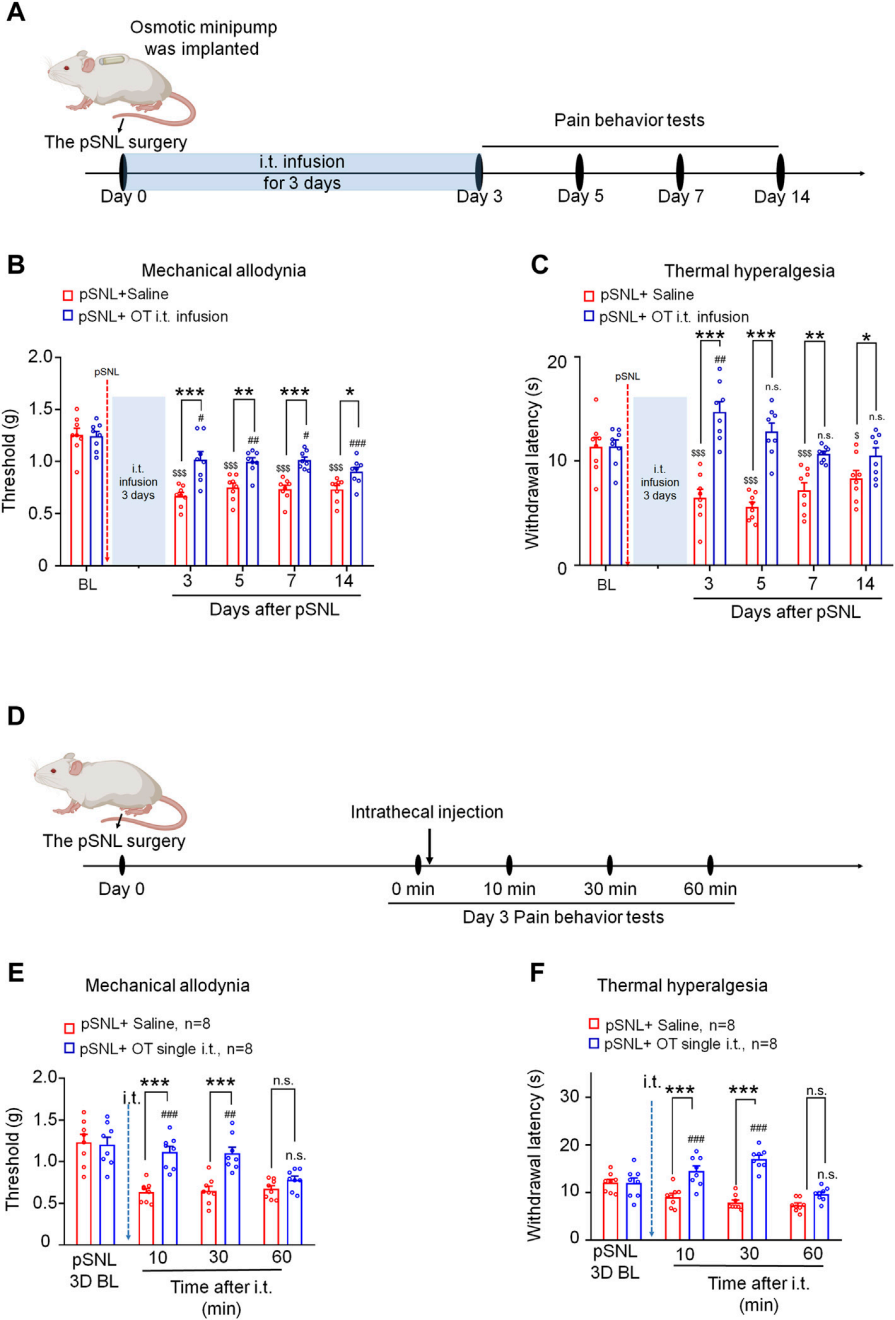
Three-day continuous intrathecal infusion, but not short-term application of OT, attenuated the establishment and development of nerve injury-induced nociceptive behaviours in pSNL mice. **(A)** A schematic of the experimental design. **(B,C)** Continuous intrathecal OT infusion (0.3 μg/100 μL) for 3 days before behavioural tests decreased pSNL-induced mechanical allodynia **(A)** and thermal hyperalgesia **(B)** for 14 days. **(D)** A schematic of the experimental design. **(E,F)** A single intrathecal OT injection (0.1 μg/10 μL) relieved pSNL-induced mechanical allodynia **(E)** and thermal hyperalgesia **(F)** in mice. Two-way repeated-measures ANOVA with group as the between-subjects factor and day/time as the within-subjects factor. Data are expressed as mean ± SEM. *p < 0.05, **p < 0.01, ***p < 0.001 OT *vs*. saline; ^$^ *p* < 0.05, ^$$$^ *p* < 0.001 *vs.* baseline; ^#^ *p* < 0.05, ^##^ *p* < 0.01, ^####^ *p* < 0.0001 *vs.* baseline.

An osmotic minipump was implanted immediately following partial sciatic nerve ligation. OT was then delivered with a flow rate of 1 μL/h for 3 days from days 0–2 after pSNL surgery. Mechanical allodynia and thermal hyperalgesia were tested at days 3, 5, 7 and 14 after pSNL surgery (Figure 1A). As shown in Figures 1B,C, infusion of OT (0.3 μg, 100 μL) for 3 days before the behavioural tests decreased nerve injury-induced nociceptive behaviours in mice. Compared with the vehicle, 3-days continuous infusion of OT increased the mechanical threshold in the von Frey test [F(1,14) = 61.57, *p* < 0.001; Figure 1B, *n* = 8] and paw withdrawal latency in the hot-plate test [F(1,14) = 50.74, *p* < 0.001; Figure 1C, *n* = 8] for 14 days, which was the longest period we tested, indicating that 3-days continuous intrathecal OT infusion may attenuate the establishment and development of nerve injury-induced neuropathic pain.

In comparison, the effect of a single injection of OT on pSNL-induced mechanical and thermal hypersensitivity was also tested on day 3 after nerve ligation, when the pain behaviours were well established (Figure 1D). Single intrathecal OT (0.1 μg/10 μL) significantly alleviated pSNL-induced mechanical allodynia [F(1,14) = 42.59, *p* < 0.001; Figure 1E] and thermal hyperalgesia [F(1,14) = 29.66, *p* < 0.001; Figure 1F] at 10 [*p* < 0.001] and 30 min [*p* < 0.001] after injection. This effect of OT was not observed at 60 min after the injection [*p* > 0.05; Figures 1E,F], indicating that the analgesic effect of a single intrathecal OT administration on nerve injury-induced pain behaviours is transient. OT at the doses used in the present study had no effect on the locomotor activity or motor coordination in mice (date not shown).

We found no significant differences between male and female mice in the analgesic effects of oxytocin [*p* > 0.05; Supplementary Figure S4].

### The Effects of 3-days OT Infusion on Nerve Injury-Induced Nociceptive Behaviours Were Mediated by Oxtrs

**FIGURE 2:**
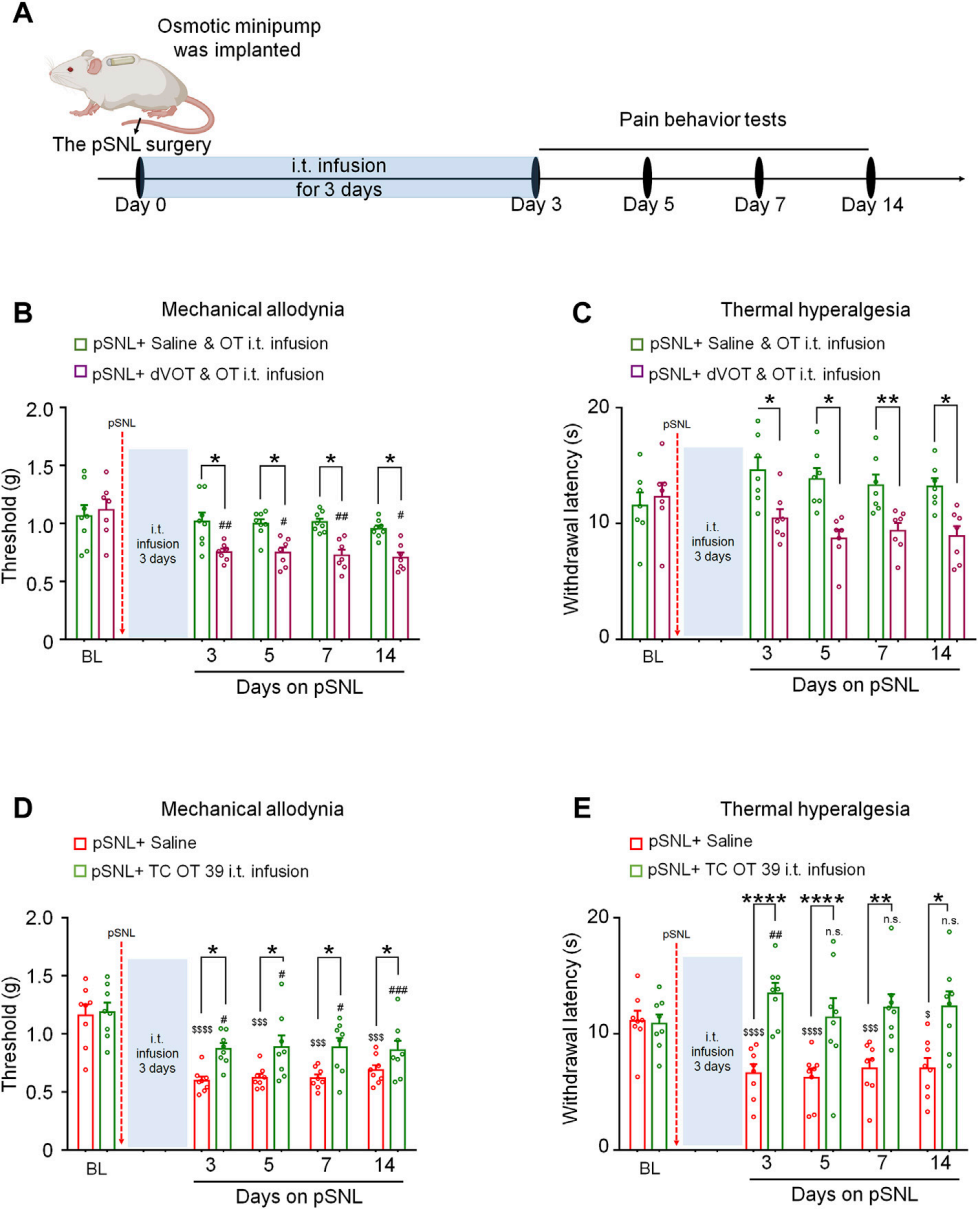
The effects of 3-days OT infusion on nerve injury-induced nociceptive behaviours were mediated by OXTRs. **(A)** A schematic of the experimental design. **(B,C)** OT’s effect on mechanical allodynia **(B)** and thermal hyperalgesia **(C)** was completely blocked by its selective antagonist, dVOT (0.3 μg/100 μL). **(D,E)** Selective OT receptor agonists, TC OT (0.3 μg/100 μL, intrathecal infusion) showed similar effects on mechanical allodynia **(D)** and thermal hyperalgesia **(E)** in pSNL mice. Two-way repeated-measures ANOVA with group as the between-subjects factor. Data are expressed as mean ± SEM. *p < 0.05, **p < 0.01, ***p < 0.001 TC OT *vs.* saline; OT *vs.* dVOT and OT. ^$$$^ *p* < 0.001, ^$$$$^ *p* < 0.0001 *vs.* baseline; ^#^ *p* < 0.05, ^##^ *p* < 0.01, ^###^ *p* < 0.001 *vs.* baseline.

To determine whether the effects of 3-days OT infusion on neuropathic pain were mediated by Oxtrs, its agonist or antagonist was administrated (Figure 2A). Co-intrathecal infusion (100 μL) of a selective Oxtr antagonist, dVOT (0.3 μg), with OT (0.3 μg) blocked the analgesic effect of OT on nerve injury-induced mechanical [F(1,13) = 25.04, *p* = 0.0002; Figure 2B, *n* = 7–8] and thermal hypersensitivity [F(1,12) = 28.92, *p* < 0.001; Figure 2C, *n* = 7]. The selective Oxtr agonists TC OT (0.3 μg/100 μL) produced significant analgesic effects which were equivalent to OT [von Frey test F(1,14) = 15.42, *p* = 0.0015; Hot-plat test F(1,14) = 29.80, *p* < 0.0001; Figures 2D,E; *n* = 8]. There results suggested that the 3-days intrathecal infusion of OT induced analgesic effect is mediated by the Oxtrs in the spinal cord.

### Three-Day Continuous Intrathecal Infusion, but Not Short-Term Application of OT, Renormalized Neuronal Chloride Equilibrium Potential in Spinal Superficial Dorsal Horn

**FIGURE 3:**
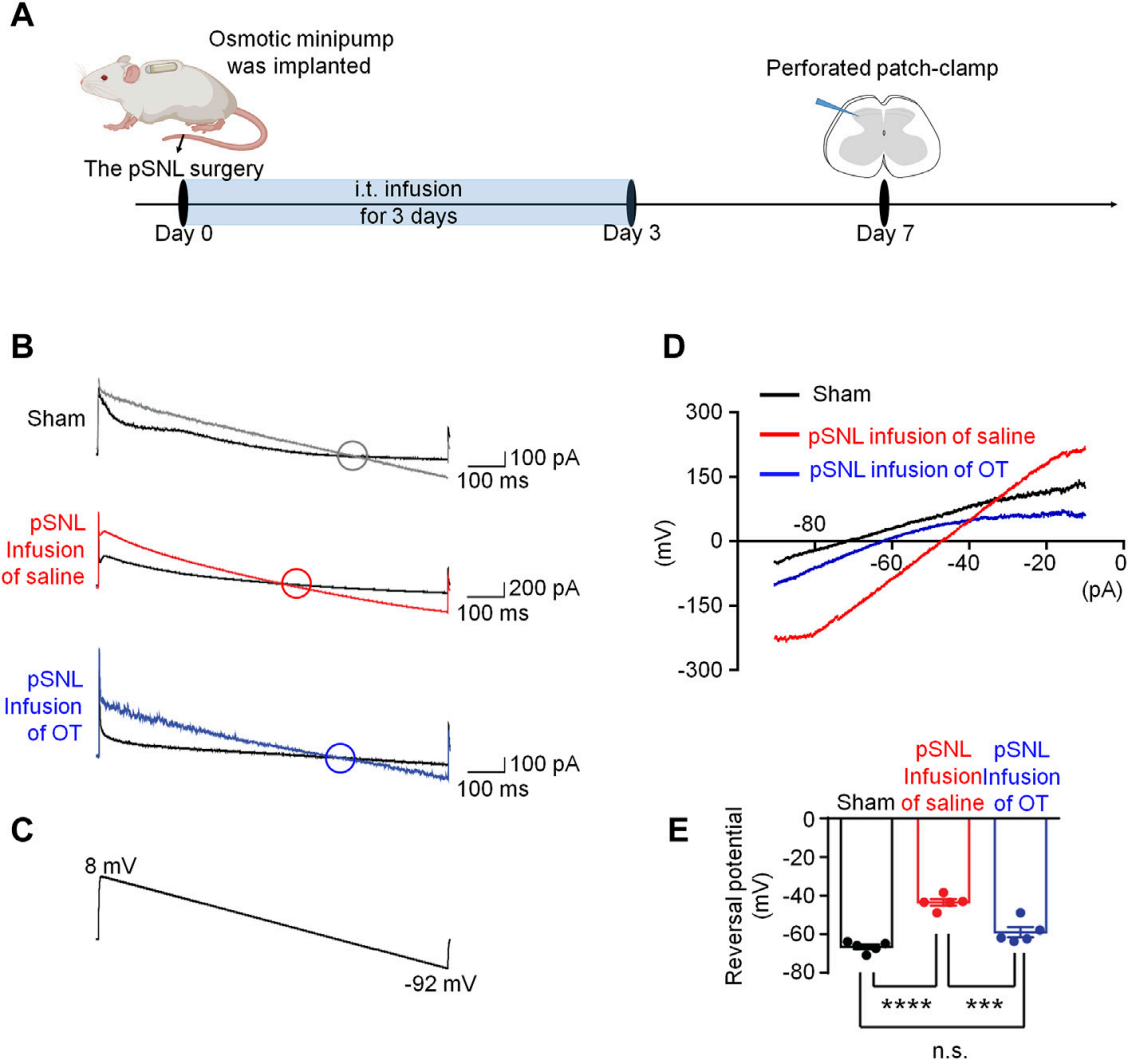
Three-day continuous intrathecal OT infusion renormalized EGABA in spinal dorsal horn. **(A)** The schematics of the electrophysiological recording. **(B,C)** As voltage ramps applied from +8 to −92 Mv **(C)**, basal and GABA-evoked currents were recorded **(B)**. **(D,E)** Representative **(D)** and statistical **(E)** reversal potential of E_GABA_ recorded from slices of sham and pSNL mice treated with continuous OT or saline. One-way ANOVA followed by Bonferroni’s post hoc test. Data are expressed as mean ± SEM. ***p < 0.001 sham *vs.* pSNL; ***p < 0.001 OT *vs.* saline infusion.

It was reported that neuronal intracellular chloride concentration was increased in the superficial dorsal horn after nerve injury (Yeo et al., 2021), we performed perforated patch-clamp recording in spinal cord slices derived from each group to investigate the effects of OT on chloride homeostasis (Figure 3A). Since GABA_A_ receptor (GABA_A_R) is the dominant chloride ion channel on the membrane of neurons in the superficial dorsal horn, GABA was puffed briefly to the recorded neuron to trigger transient chloride influx or efflux.

As voltage ramps were applied from +8 to −92 mV (Figure 3C), the GABA-evoked currents were recorded to evaluate chloride equilibrium potential (*E*_*Cl*_^*-*^). These currents were completely blocked by a selective GABA_A_R antagonist, bicuculline (10 μM), confirming that they were mediated by GABA_A_R (data not shown). The *E*_*Cl*_^*-*^ in sham mice was −66.68 ± 1.22 mV (Figures 3B–E, *n* = 5–6, 3 mice per group), whereas that value in pSNL mice shifted to a more positive value of −43.54 ± 1.67 mV [ *p* < 0.001 *vs*. sham group; F(2,12) = 36.26, *p* < 0.001; Figures 3B–E, *n* = 5 from 3-4 mice]. Continuous intrathecal infusion of OT reversed the value of *E*_*Cl*_^*-*^ to −59.02 ± 2.69 mV, which was much closer to that of the sham mice [*p* > 0.05 *vs*. sham; Figures 3B–E, *n* = 5 from 3-4 mice], suggesting that 3-days infusion of OT was able to restore [Cl^−^]_i_ in pSNL mice.

**FIGURE 4:**
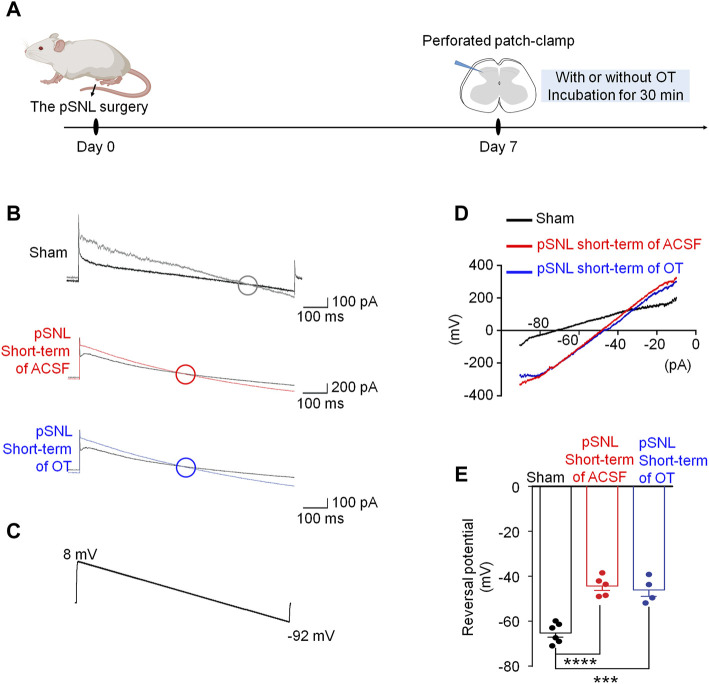
Short-term OT incubation failed to renormalize EGABA in spinal dorsal horn. **(A)** The schematics of the electrophysiological recording. **(B,C)** As voltage ramps were applied from +8 to −92 Mv **(C)**, basal and GABA-evoked currents **(B)** were recorded. **(D,E)** The reversal potential of E_GABA_ recorded from slices of naïve and pSNL mice incubated with OT or saline. One-way ANOVA followed by Bonferroni’s post hoc test. Data are expressed as mean ± SEM. ***p < 0.001, ****p < 0.0001 naïve *vs.* pSNL incubated with saline or OT.

In comparison, we also recorded the *E*_*Cl*_^*-*^ using the spinal cord slices incubated with saline or OT for 30 min (short-term application, Figure 4A), and the reversal potentials were −44.34 ± 2.91 mV and −46.10 ± 3.10 mV, respectively [*p* > 0.05 *vs.* saline; F(2,12) = 31.71, *p* < 0.0001; Figures 4B–E]. Incubation of the spinal cord slices with OT for a relatively short time failed to restore the value of *E*_*Cl*_^*-*^ in pSNL mice, suggesting that the effect of OT on *E*_*Cl*_^*-*^ required relatively long-term application.

### Three-Day Continuous Intrathecal OT Infusion Upregulated Spinal KCC2 Expression

**FIGURE 5:**
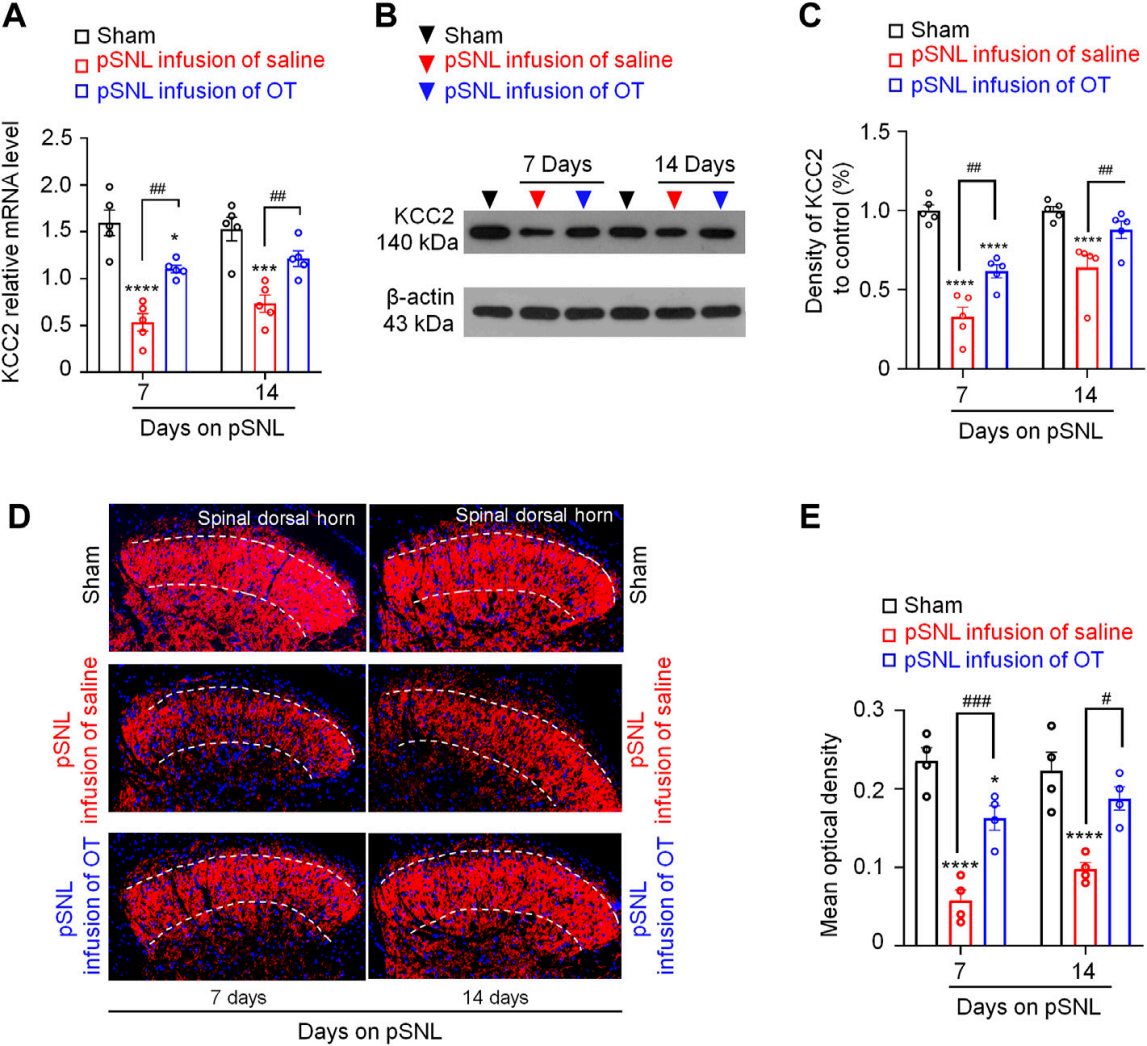
Three-day continuous intrathecal OT infusion increased KCC2 expression in the spinal dorsal horn in pSNL mice. **(A)** Continuous intrathecal OT infusion increased spinal KCC2 mRNA on days 7 and 14 after pSNL. **(B,C)** Continuous intrathecal OT infusion upregulated spinal KCC2 protein levels on days 7 and 14 after pSNL. **(B)** Representative western blots of KCC2 and the loading control (β-actin) are presented for each group. **(D)** Representative image shows the staining of KCC2 (red) in naïve mice and in pSNL mice treated with saline or OT. DAPI was used to stain the cell nuclei (blue) **(E)** The intensity of KCC2 staining. One-way repeated measures ANOVA was used to analyse differences across days within each group. Simple effects ANOVA was used to confirm differences between groups at each time point. Data are expressed as mean ± SEM. ^#^ *p* < 0.05, ^##^ *p* < 0.01, ^###^ *p* < 0.001 *vs.* saline; ***p < 0.001, ****p < 0.0001 *vs.* sham.

Given that the shift of *E*_*Cl*_^*-*^ in pSNL animals may be due to depressed function of KCC2, we analysed the transcriptional and expression levels of KCC2 in the spinal cord. Compared with the sham group, quantitative PCR data revealed a significant decrease in spinal *Kcc2* mRNA levels at both days 7 and 14 after pSNL surgery [*p* < 0.001 vs. sham; F(2,16) = 3.818, *p* = 0.0441; Figure 5A, *n* = 5 per group]. Intrathecal infusion of OT increased spinal *Kcc2* mRNA levels in pSNL mice compared with saline group [*p* < 0.01; F(2,16) = 3.818, *p* = 0.0441; Figure 5A, *n* = 5 per group].

Western blotting data also showed that nerve injury-induced a significant decrease in the protein levels of KCC2 in the spinal dorsal horn at days 7 and 14 after pSNL surgery [*p* < 0.0001 *vs*. sham; F(2,16) = 8.982, *p* = 0.0024; Figures 5B,C, *n* = 5 per group]. Intrathecal infusion of OT restored the protein levels of KCC2 but did not completely reverse this decrease [*p* < 0.01 *vs.* saline; F(2,16) = 8.982, *p* = 0.0024; Figures 5B,C, *n* = 5 per group]. Immunohistochemistry (IHC) of spinal slices from laminae II further supported the western blotting data, which showed that the KCC2 signal was widely expressed throughout the spinal dorsal horn in sham mice (Figure 5D). Nerve injury-induced a reduction in KCC2 expression at days 7 and 14 after pSNL surgery [*p* < 0.0001 *vs*. sham; F(2,12) = 8.119, *p* = 0.0059; Figures 5D,E]. Infusion of OT reversed this reduction [*p* < 0.01 *vs.* sham; F(2,12) = 8.119, *p* = 0.0059; Figures 5D,E, *n* = 4 per group] to some extent.

### Oxtrs Are Functionally Expressed in Inhibitory Interneurons and OT Enhanced GABAergic Inhibitory Transmission Through Activation of Oxtrs in the Superficial Dorsal Horn

**FIGURE 6:**
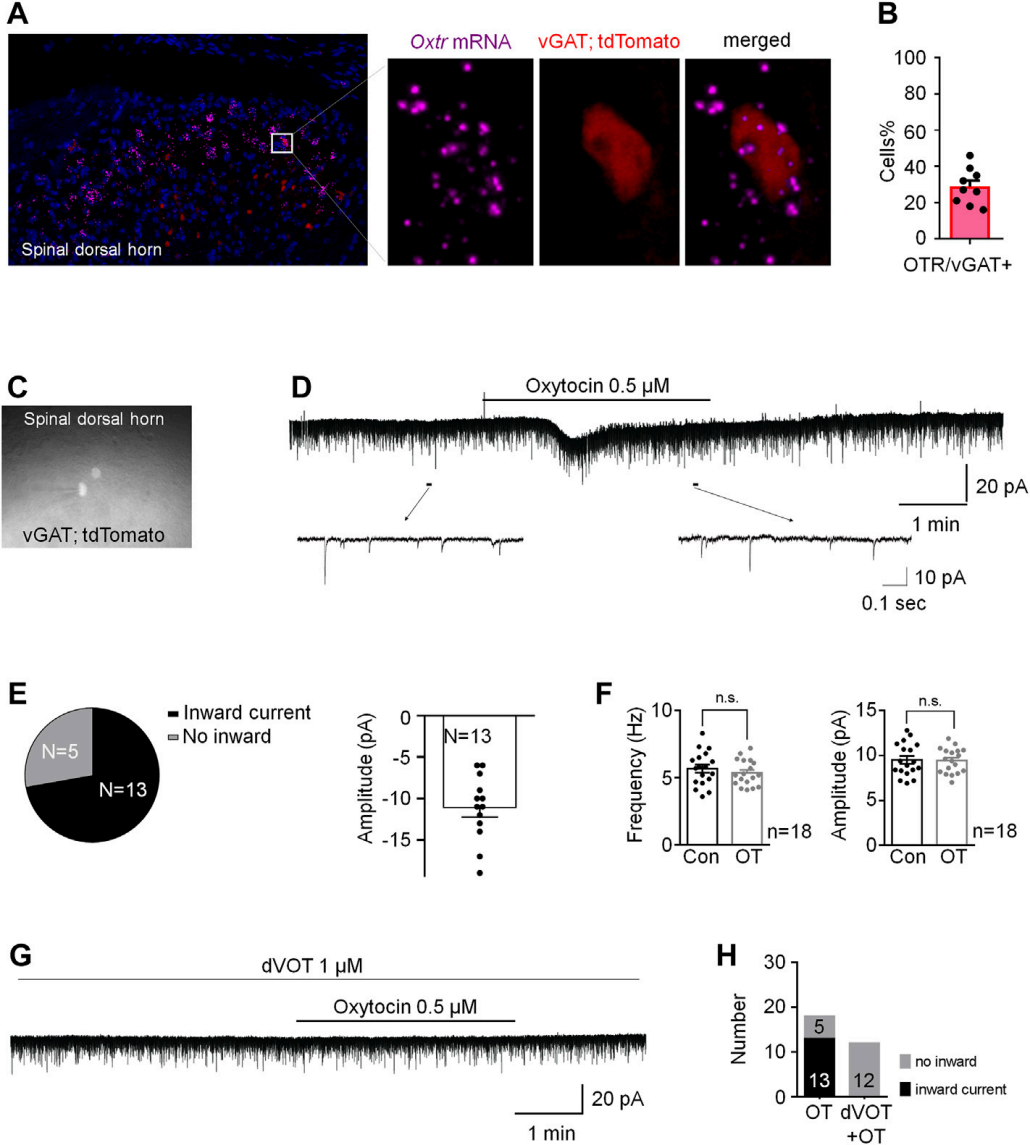
OT produced an inward current in vGAT+ neurons through activation of Oxtrs in the superficial dorsal horn. **(A)** RNAscope showed that Oxtrs (pink) were expressed on the inhibitory neurons (red) in the spinal dorsal horn. Co-expression of a sample inhibitory neuron (red) and the puncta representing Oxtrs (pink) in the enlarged image. DAPI was used to stain the cell nuclei (blue). **(B)** percentage of Oxtrs expressed in the vGAT + neurons. **(C)** The vGAT^**+**^ interneurons in the superficial dorsal horn. **(D,E)** OT perfusion produced an inward current in 72% recorded vGAT + neurons (*n* = 18). **(F)** The frequency and amplitude of spontaneous EPSCs in all examined vGAT + neurons. Paired *t*-test. Data are expressed as mean ± SEM. **(G,H)** Selective Oxtr antagonist dVOT (1 μM) blocked OT induced inward currents in all recorded vGAT positive interneurons in the superficial dorsal horn (*n* = 12).

To further explore the underlying mechanism of OT on the regulation of *E*_*Cl*_^*-*^, we performed a novel *in situ* hybridization assay (RNAscope) to investigate the feature of Oxtr mRNA expression. Firstly, we used a novel *in situ* hybridization assay (RNAscope) to detect the properties of Otxr mRNA distributions in the superficial dorsal horn. As shown in Supplementary Figure S2, Oxtrs mRNA (white) were not expressed on microglia (green) and astrocytes (red), suggesting that majority of Oxtrs are located in the neurons. To test whether that Oxtrs were expressed on the inhibitory neurons in the spinal dorsal horn. Spinal cord slices derived from the vGAT-tdTomato mice were used, in which the inhibitory neurons were visualized by red fluorescence. As shown in Figures 6A,B, about 30% of vGAT + neurons (inhibitory neurons) expressed Oxtrs mRNA signalling in the in the spinal dorsal horn. Oxtr mRNAs were also found expressed in vGAT negative interneurons in the superficial dorsal horn.

We then performed whole-cell voltage clamp on the vGAT positive interneurons in the superficial dorsal horn. About 72% recorded vGAT^+^ neurons (*n* = 18) produced an inward current when OT (0.5 μM) was perfused for 3 min at the V_H_ of −70 mV with an average of −10.40 ± 1.27 pA (upper trace in Figures 6C–E), but OT did not change the frequency and amplitude of spontaneous EPSCs in all of the examined vGAT^+^ neurons [*t*-test, *p* = 0.0663, t (34) = 1.963 for frequency; *p* = 0.6311, t (34) = 0.4890 for amplitude; Figure 6F]. In the presence of the Oxtr antagonist dVOT (1 μM), OT failed to induce an inward current in all recorded vGAT positive interneurons in the superficial dorsal horn (Figures 6G,H, *n* = 12). In comparison, OT perfusion produced an inward current in 38% recorded vGAT negative neurons (Supplementary Figures 3B,C, *n* = 13).

**FIGURE 7:**
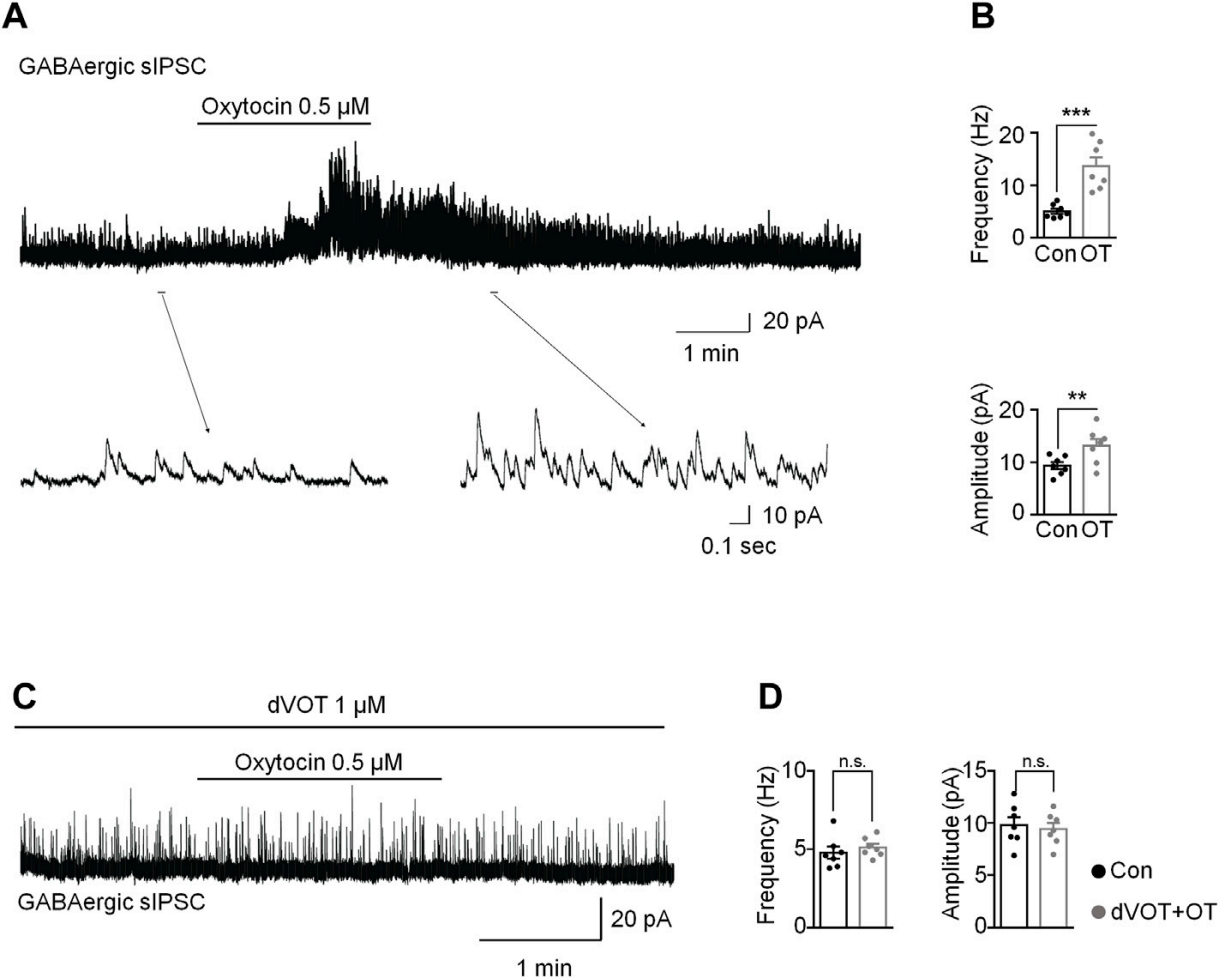
OT enhanced GABAergic inhibitory transmission through activation of OXTRs in the superficial dorsal horn. **(A,B)** OT perfusion increased the frequency and amplitude of spontaneous GABAergic IPSCs. **(C,D)** The selective Oxtr antagonist dVOT blocked OT-enhanced GABAergic spontaneous transmission. Paired *t*-test. Data are expressed as mean ± SEM. **p < 0.01, ***p < 0.001 *vs.* control.

Due to OT produced inward currents in some vGAT positive interneurons, we tested the effects of OT on GABAergic transmission in the spinal cord in the presence of a glycine-receptor antagonist, strychnine (1 μM). OT (0.5 μM) perfusion for 3 min increased the frequency and amplitude of spontaneous GABAergic IPSCs at the V_H_ of 0 mV from 5.02 ± 0.49 Hz to 13.61 ± 1.72 Hz and 9.40 ± 0.68 pA to 13.17 ± 1.30 pA, respectively (*t*-test, *p* = 0.0009, t (12) = 6.026 for frequency; *p* = 0.0080, t (12) = 3.899 for amplitude; *n* = 7; Figures 7A,B). Expectedly, OT enhanced GABAergic spontaneous transmission was total blocked by pre-treatment with a selective Oxtr antagonist, dVOT (1 μM, *p* = 0.2498, t (12) = 1.274 for frequency; *p =* 0.2987, t (12) = 1.138 for amplitude; *n* = 7; Figures 7C,D).

## Discussion

In this study, we demonstrated that three days of continuous intrathecal OT infusion alleviated subsequent pain behaviours for 14 days, whereas a single OT injection induced a transient analgesia for 30 min in mice. Supporting this behavioural finding, only continuous intrathecal infusion, but not short-term incubation of OT, reversed the nerve injury-induced depolarizing shift in Cl^−^ reversal potential, which was mediated by improving the function and expression of spinal K^+^-Cl^-^ cotransporter 2 (KCC2). This result suggests that only continuous use of OT may reverse the pathological changes caused by nerve injury, thereby mechanistically blocking the establishment and development of pain.

Pain is a multidimensional experience that includes not only nociceptive and nocifensive components but also emotional-affective and cognitive components. As OT is involved in a wide range of behaviours, it is a promising target for the therapeutic pain intervention. The number of studies supporting that OT has antinociceptive effects grows steadily. Animal studies in particular have delivered robust evidence supporting this idea. Unfortunately, these findings have not been translated into therapeutics. We believe at least two issues have hampered the clinical use of OT. One is the poorly defined mechanisms of action of OT, and the other is difficulty with OT delivery to the central nervous system. Here, we adopted intrathecal drug delivery technique to administer OT centrally in nerve injured mice to understand how continuous use of OT acts on the pathological changes caused by nerve injury.

As the results showed in this study, continuous intrathecal OT infusion for three days alleviated subsequent pain behaviours induced by nerve injury. It is noteworthy that the pSNL mice that received the OT perfusion in advance showed continuous relief in pain behaviours for 14 days, which was as long as we tested, although the OT perfusion has stopped during behavioural tests. This result suggested that continuous intrathecal OT infusion may attenuate the establishment and development of nerve injury-induced neuropathic pain. In comparison, a single intrathecal injection of OT in intact or neuropathic pain model mice only induced a transient analgesia for 30 min. The short-term analgesic effect of a single administration of OT revealed in this study was compatible with the results derived from other pain models. For example, Yu found that the duration of analgesia of OT was within 1 hour in inflammatory pain (Yu et al., 2003), and Yang reported that the effects of intraventricular or intrathecal injection of OT lasted about 30 min in intact rats (Yang et al., 2007).

We also observed that intrathecal OT infusion not only reverse thermal hyperalgesia but induces analgesia one day after OT continuous infusion. A single injection of OT also showed an analgesia effect in the hotplate test 30 min after injection. This analgesic effect of OT may be related to presynaptic TRPV1 inhibition in the spinal cord (Sun et al., 2018). Since we found no significant differences between male and female mice in the analgesic effects of OT on day 3 after pSNL surgery (Supplementary Figure S4).We conducted the experiments using male mice in the present study. However, we cannot rule out sex differences in the effect of intrathecal OT infusion.

All the behavioural tested were conducted within 14 days after the pSNL surgery. Since inflammatory component existed post-surgery, the current results cannot rule out that anti-inflammatory mechanisms are involved in the analgesic effect of OT.

OT plays its effects by activating OT receptors, which belongs to the G protein-coupled receptor superfamily, together with the three structurally related arginine-vasopressin (AVP) receptors (V1aR, V1bR and V2R), forms a small receptor sub-family. All of these receptors bind to OT albeit with different affinities and eliciting different responses. Selective activating OXTRs by its agonist, TC OT produced significant analgesic effects which were equivalent to OT, whereas antagonizing OXTR by its antagonist, dVOT blocked the analgesic effect of OT in pSNL mice, indicating that intrathecal OT infusion induced analgesic effect is mediated by the OXTRs in the spinal cord.

The current understanding of mechanisms underlying OT analgesia is mainly based on studies using single or multiple injections of OT. The acute analgesic mechanisms of OT involve GABA, potassium channels, sodium channels and TRPV channels (Breton et al., 2008; Jiang et al., 2014). Little is known about the actions of continuous, relatively long-term OT administration on pain processing. It is proposed that nerve injury causes an imbalance between excitatory and inhibitory control in the nervous system, which is partially caused by a loss of inhibition in the dorsal horn of the spinal cord and which is in turn responsible for neuropathic pain (Kuner, 2010). The broken of neuronal intracellular Cl^−^ homeostasis is a major cause for the loss of inhibition in spinal dorsal horn. In order to investigate the underlying mechanisms of continuous intrathecal OT infusion on pain processing, we tested whether they block neuropathic pain by preventing the disruption of the intracellular Cl^−^ homeostasis in the spinal superficial dorsal horn, a key region in nociceptive information transmission; and whether it is mediated by restoring the KCC2 expression and function.

Firstly, we found that the chloride equilibrium potential (*E*_*Cl*_^*-*^) in pSNL mice was significantly shifted to a more positive value by using whole-cell patch-clamp technique, indicating an elevated level of [Cl^−^]_i_ in pSNL animals. The result was consistent with the previous finding that neuronal intracellular chloride concentration was increased in the superficial dorsal horn after nerve injury (Yeo et al., 2021). Only 3-days continuous intrathecal infusion, but not a short-term incubation of OT, restored the value of *E*_*Cl*_^*-*^, suggesting that only continuous intrathecal OT infusion was able to restore [Cl^−^]_i_. Considering neuronal chloride homeostasis plays important role in pain processing, this result indicated that continuous oxytocin infusion renormalized neuronal chloride homeostasis to attenuates neuropathic pain.

KCC2 (Cl^−^ extrusion) and NKCC1 (Cl^−^ uptake) are the most important chloride transporters in cortical neurons and therefore represent the main regulators of chloride homeostasis (Kaila, 1994; Delpire, 2000). The elevated level of [Cl^−^]_i_ in neurons suggested a downregulation of KCC2 or an upregulation of NKCC1. Only continuous intrathecal infusion, but not a short-term incubation of OT, restored chloride homeostasis, and suggested the altered function of KCC2 or NKCC1 in pSNL animals.

Since it is reported that lack of Oxtr in neurons affects specifically KCC2 without impairing NKCC1 (Leonzino et al., 2016), we then used quantitative PCR, western blotting and immunohistochemistry to test whether the continuous intrathecal OT infusion upregulated spinal KCC2 expression and rescued the decrease in KCC2 expression by nerve injury. As the results showed, nerve injury induced a significant decrease in the expression levels of KCC2 after pSNL. Intrathecal infusion of OT restored the expression levels of KCC2 in the spinal dorsal horn.

Coull and his colleagues have shown that the inhibitory control in GABAergic neurons in the spinal dorsal horn can be lost when KCC2 activity is impaired, which can eventually lead to neuropathic pain (Coull et al., 2003). In mature central neurons, KCC2 is responsible for the low [Cl^−^]_i_ that forms the basis for hyperpolarizing GABA_A_ receptor-mediated responses. Changes in KCC2 function and expression have been observed under various physiological and pathophysiological conditions. Nerve ligation often tends to decrease spinal KCC2 expression, which contributes to the development of neuropathic pain. Nerve injury-induced brain-derived neurotrophic factor (BDNF) release may account for the reduction in KCC2 (Kitayama, 2017). Therefore, it is indicated that spinal KCC2 expression is responsible for the development and maintenance of neuropathic pain. Continuous infusion of OT may attenuate the development and maintenance of neuropathic pain by restoring the alternations of KCC2.

As a small polypeptide, oxytocin is rapidly broken down in the gastrointestinal system. It has a very short half-life of 3–5 min in the blood. Although the half-life of OT is much longer in CSF (∼28 min) than in the blood, it is known to penetrate the blood brain barrier only sparingly (Kang and Park, 2000), making oral or parenteral administration untenable. Thus, human OT effects on pain sensitivity have most frequently been investigated using the intranasal administration route. However, there are many constraints to the intranasal application of this neuropeptide that might contribute to the rather inconsistent findings in human studies. In one study, the elevation of OT levels in the CSF was observed only in one out of the six macaques that received intranasal OT (Lee et al., 2018). In 1984, Penn and Kroin introduced intrathecal administration of baclofen in humans to alleviate spasticity in severe cases (Penn and Kroin, 1984). Since then, intrathecal drug delivery has become an important treatment option for individuals with severe spasticity, dyskinetic cerebral palsy, stiff-man syndrome, and chronic pain (Penn and Mangieri, 1993; Saval and Chiodo, 2008; Eek et al., 2018). Drugs can be administered *via* an intrathecal route that allows for the placement of the medication in close proximity to the target receptors so that a much lower dose is needed. By using continuous intrathecal delivery, a steady drug concentration can be maintained within the central nervous system (Mathur et al., 2014). In a long-term (>10 years) clinical study where Baclofen was administrated intrathecally, patients reported a high level of treatment and life satisfaction (McCormick et al., 2016). These findings provide novel evidence relevant for advancing understanding of the effects of continuous OT administration on the pathophysiology of pain.

Many factors may mediate OT-induced KCC2 upregulation. It has been reported that BDNF may be the cause of the reduction in KCC2. As a neurotrophic factor, BDNF is produced and secreted mainly by microglia (Fujita et al., 2008). This study showed that Oxtrs were mainly expressed in the neurons, but not glia cells. So we speculate that OT did not upregulated of KCC2 through BDNF. In this study, we also found that OT enhanced GABAergic inhibitory transmission through activation of Oxtrs in the spinal dorsal horn, which may help us to understand the mechanisms underlying continuous OT’s action on KCC2. We first confirmed by RNAscope that Oxtr mRNA was expressed on some of the inhibitory neurons in the spinal dorsal horn, although it was also observed in vGAT negative neurons. We then performed whole-cell voltage clamps to record the spontaneous EPSC in the inhibitory interneurons. OT perfusion produced an inward current without affecting the frequency and amplitude of spontaneous EPSCs in the inhibitory neurons. This result suggested that OT produced a depolarization in some inhibitory neurons without affecting glutamatergic transmission. As a result of the depolarization of inhibitory neurons, GABA may be released, which was further confirmed by the finding that OT enhanced GABAergic spontaneous transmission by increasing both the frequency and amplitude of spontaneous GABAergic IPSCs. These effects of OT on GABAergic inhibitory transmission were completely blocked by perfusion of a selective OTXR antagonist, dVOT. Ganguly et al. reported that GABAergic activity drove the increase in the level of KCC2 mRNA in mature neurons (Ganguly et al., 2001). Heubl et al. further demonstrated that enhancing GABA_A_R-mediated inhibition confines KCC2 to the plasma membrane, while antagonizing inhibition reduces KCC2 surface expression by increasing the lateral diffusion and endocytosis of the transporter. This mechanism utilizes Cl^−^ as an intracellular secondary messenger and is dependent on the phosphorylation of KCC2 at threonines 906 and 1007 by the Cl^−^-sensing kinase WNK1. Taken together, we hypothesis that OT up-regulated KCC2 in neuropathic pain through the activation of GABAergic inhibitory transmission. However, this hypothesis is based on the transient actions of OT on the inhibitory neurons. Long-term application (3-days infusion) of OT may have many consequences on receptor binding, trafficking and expression. Therefore, we cannot rule out that the effect of OT on inhibitory neurons may be different when applied for a relatively long time, and that there are other mechanisms involved in OT-induced upregulation of KCC2.

## Conclusion

To conclude, this study used an intrathecal delivery technique to demonstrate that continuous intrathecal OT infusion attenuated the subsequent establishment and development of nerve injury-induced neuropathic pain and renormalized neuronal chloride homeostasis *via* upregulation of KCC2 expression and function, which may be caused by OT-induced activation of GABA inhibitory transmission. These findings provide novel evidence relevant for advancing the understanding of the effects of continuous OT administration on the pathophysiology of pain.

## Data Availability

The raw data supporting the conclusion of this article will be made available by the authors, without undue reservation.
