# Supplementary figures and images for: Three-Day Continuous Oxytocin Infusion Attenuates Thermal and Mechanical Nociception by Rescuing Neuronal Chloride Homeostasis via Upregulation KCC2 Expression and Function
Source: Front Pharmacol. 2022 Mar 24;13:845018. doi: 10.3389/fphar.2022.845018 (PMC8988046; doi:10.3389/fphar.2022.845018)

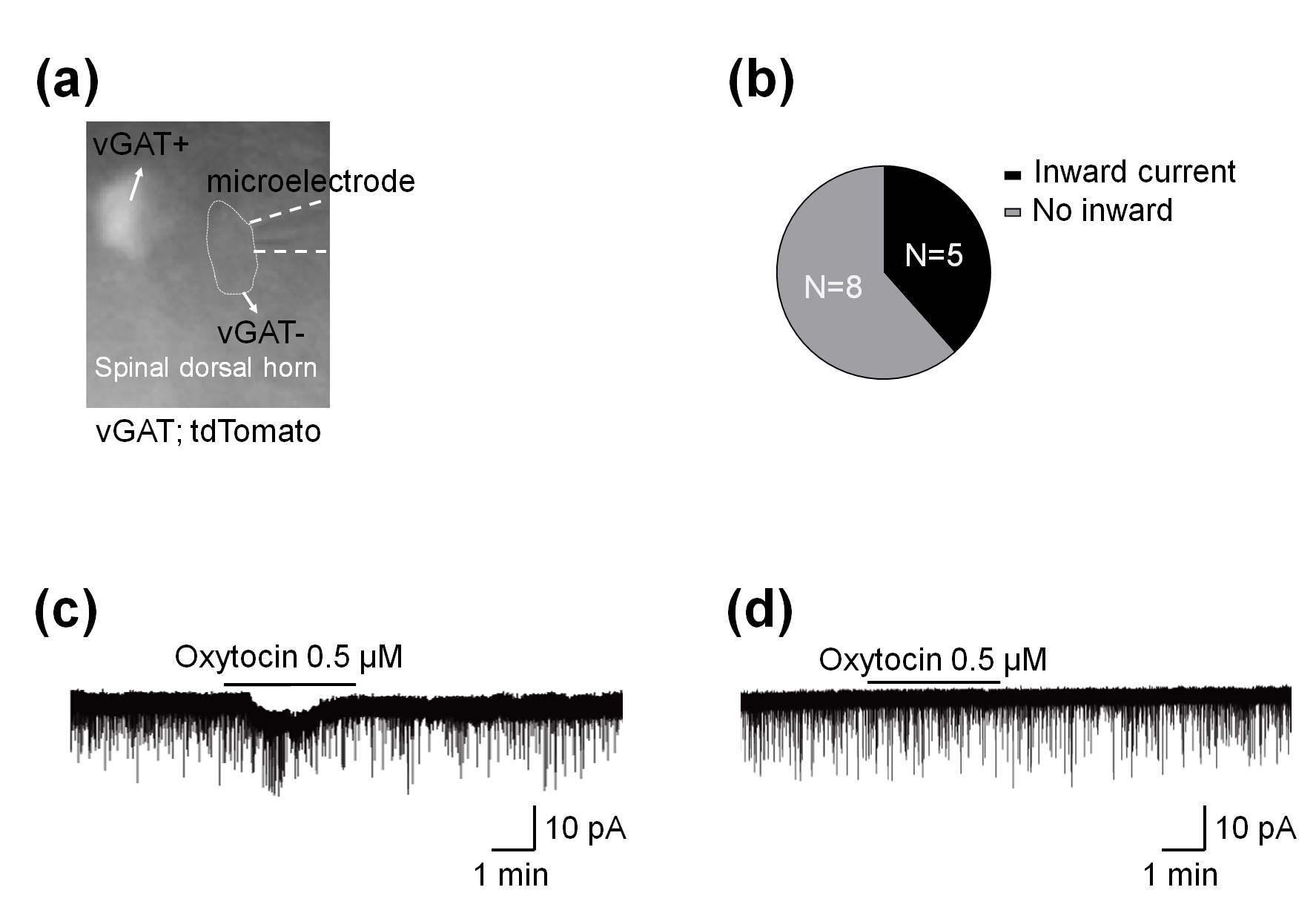

Supplement: Supplementary file 1 [file Figure10.TIF]

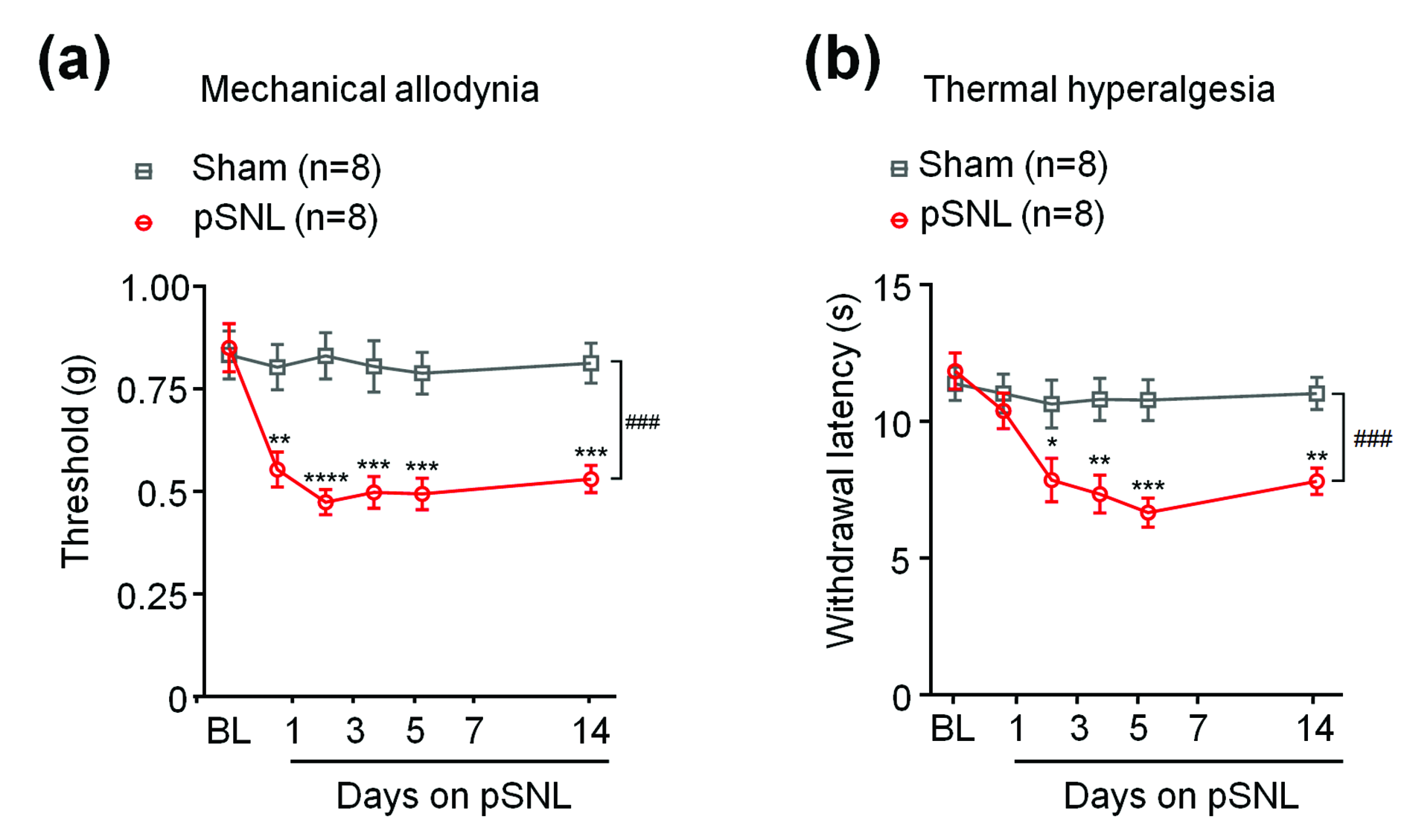

Supplement: Supplementary file 2 [file Figure8.TIF]

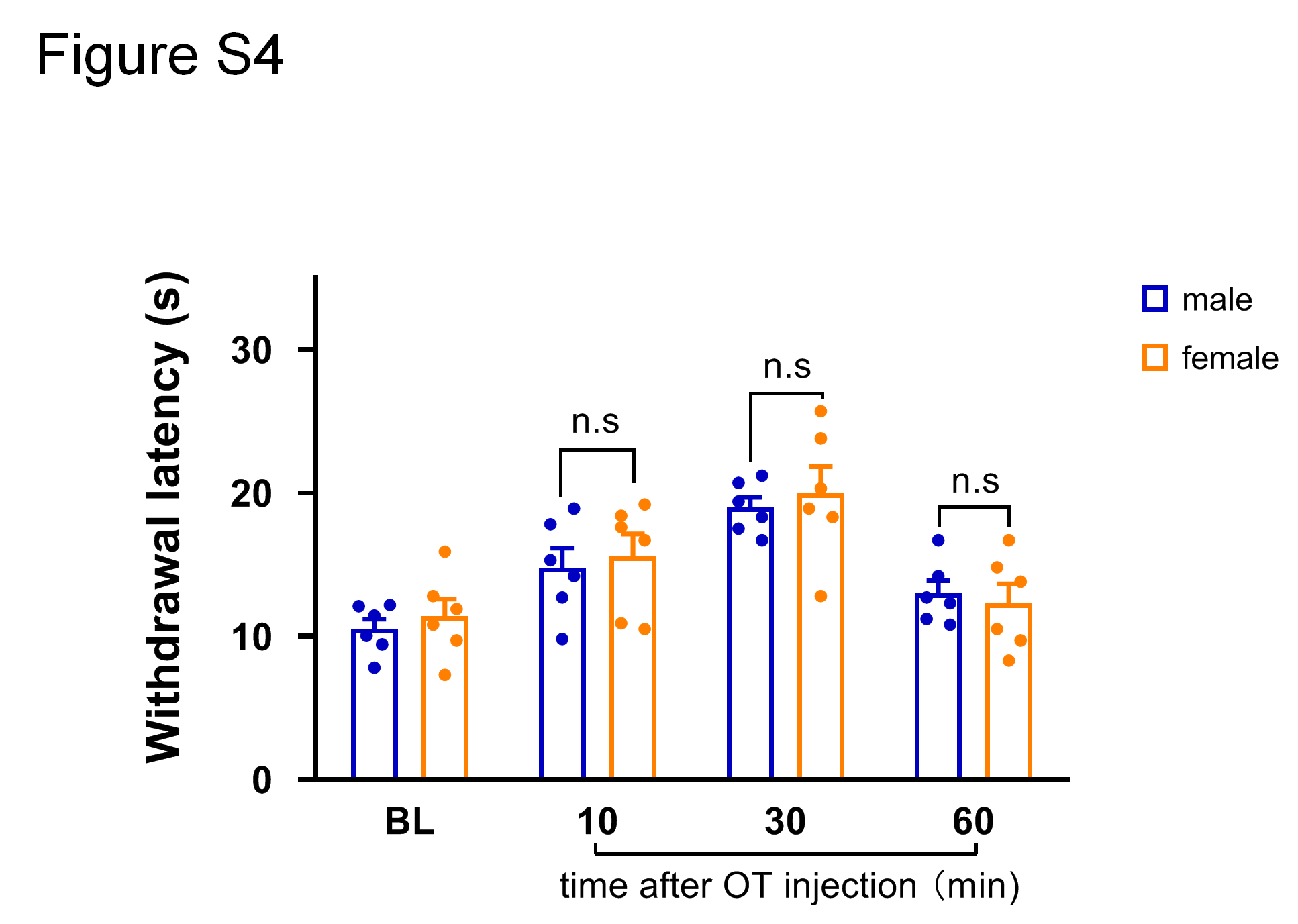

Supplement: Supplementary file 3 [file Figure11.TIF]

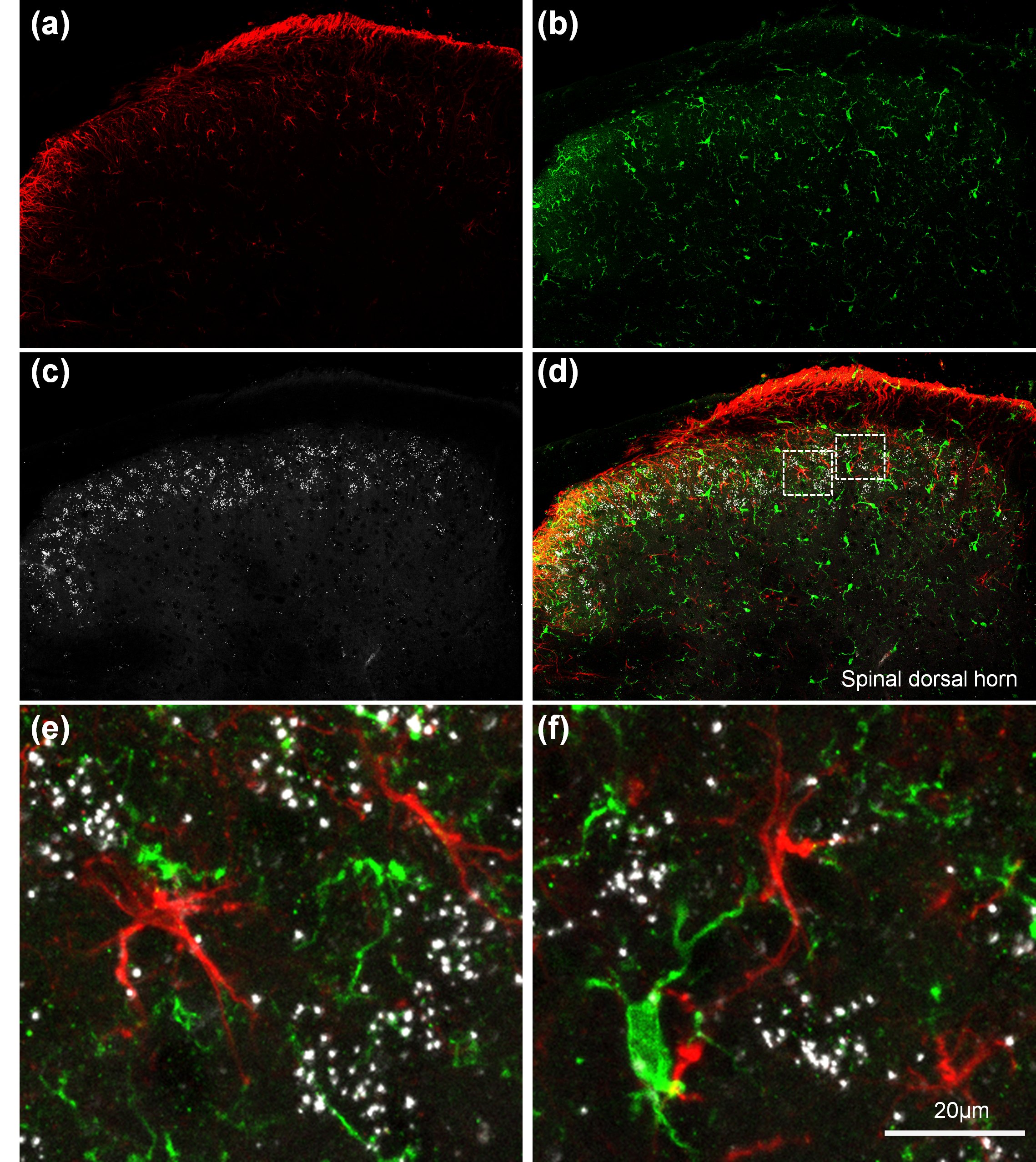

Supplement: Supplementary file 4 [file Figure9.TIF]

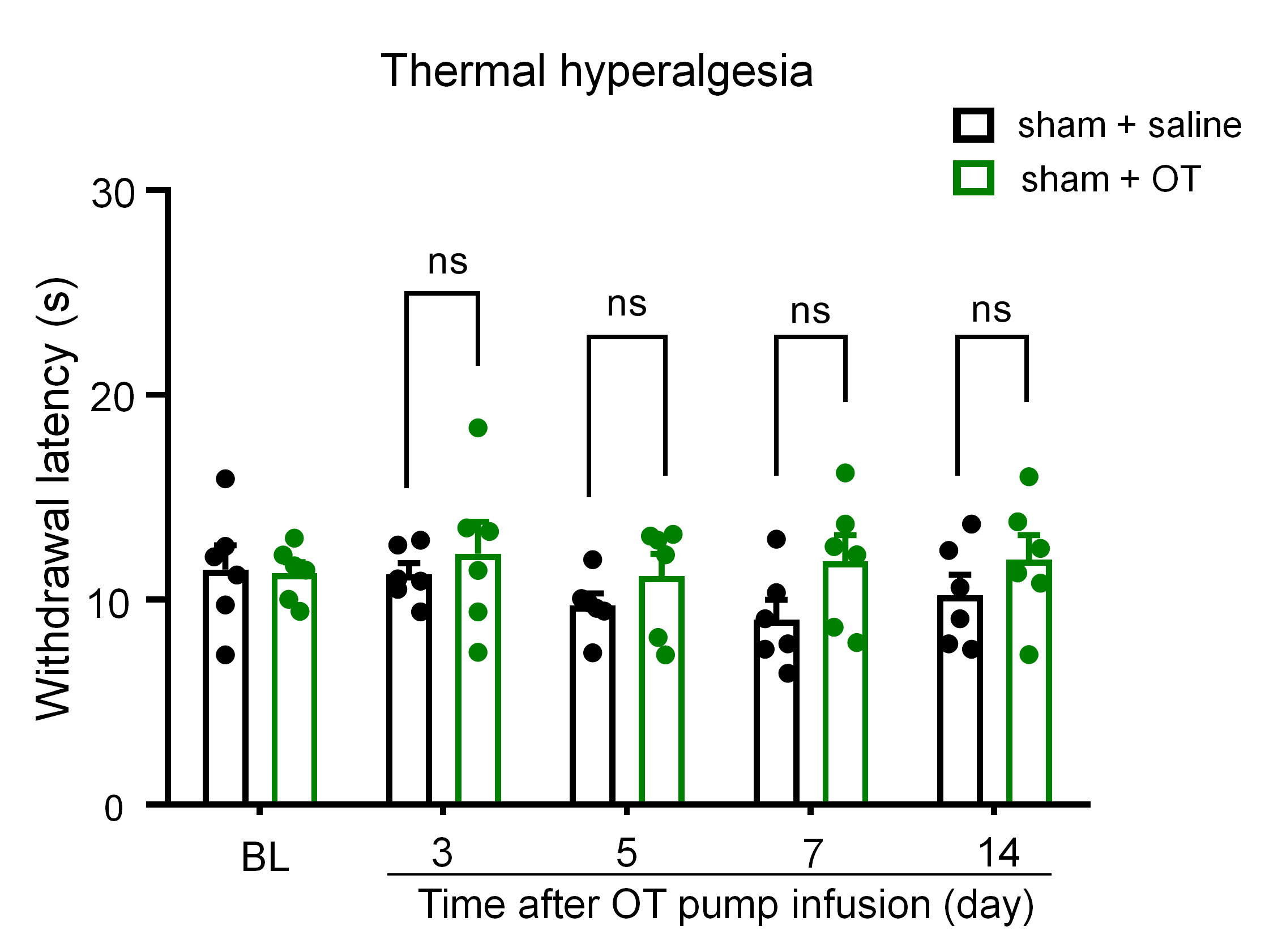

Supplement: Supplementary file 5 [file Figure13.TIF]

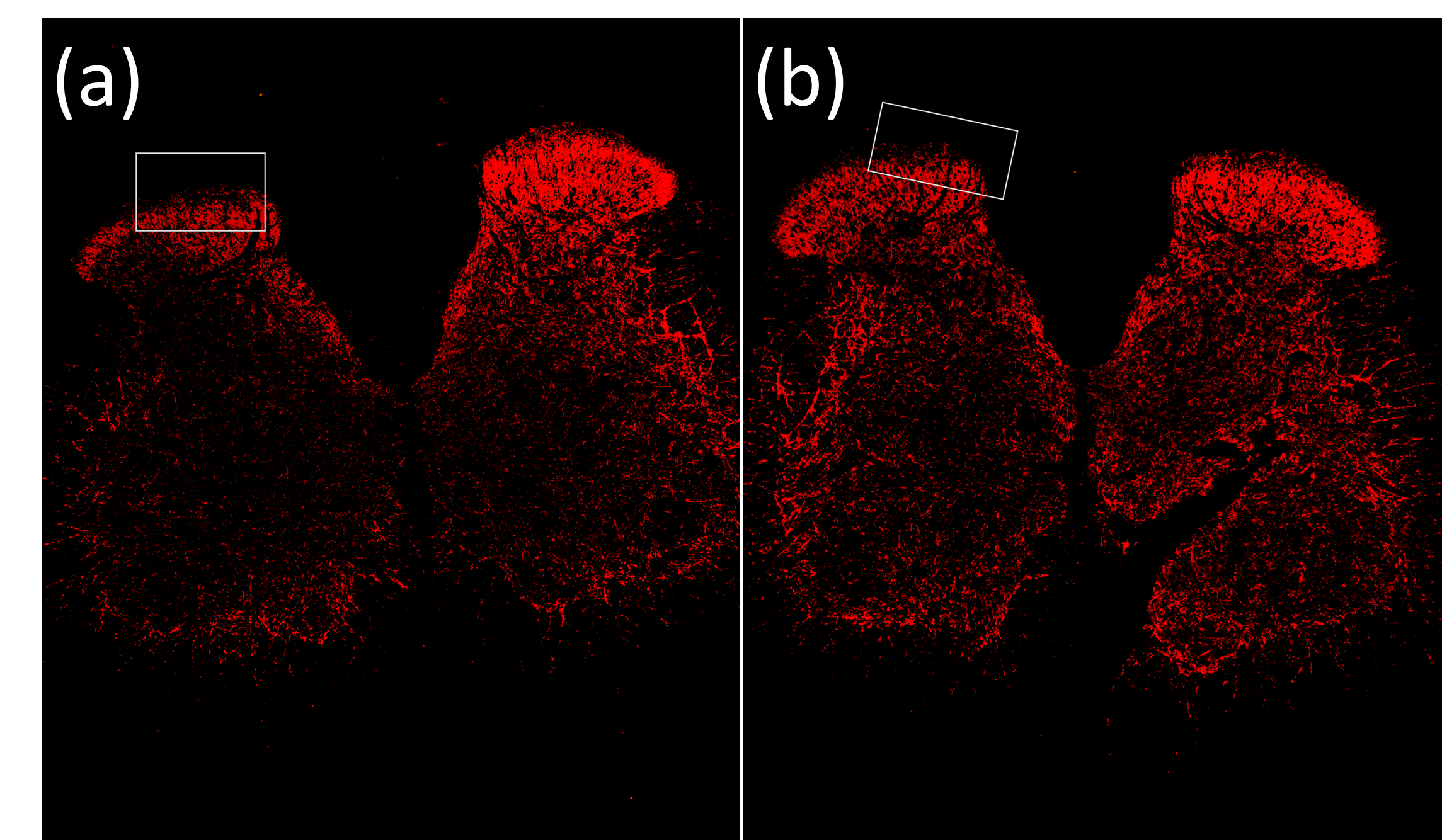

Supplement: Supplementary file 6 [file Figure12.TIF]
